# New PCR Assays for the Identification of *Fusarium verticillioides*, *Fusarium subglutinans*, and Other Species of the *Gibberella fujikuroi* Complex

**DOI:** 10.3390/ijms13010115

**Published:** 2011-12-23

**Authors:** Carla Bertechini Faria, Camila Agnes Lumi Abe, Cleiltan Novais da Silva, Dauri José Tessmann, Ione Parra Barbosa-Tessmann

**Affiliations:** 1 Department of Biochemistry, State University of Maringá, Av. Colombo, 5790, 87020-900, Maringá, PR, Brazil; E-Mails: cbertechini.faria@gmail.com (C.B.F.); camilaagneslumiabe@gmail.com (C.A.L.A.); 2 Department of Agronomy, State University of Maringá, Av. Colombo, 5790, 87020-900, Maringá, PR, Brazil; E-Mails: cleiltan@gmail.com (C.N.S.); djtessmann@uem.br (D.J.T.)

**Keywords:** PCR, *gaoB* gene, molecular identification, *Gibberella fujikuroi*

## Abstract

*Fusarium verticillioides* and *Fusarium subglutinans* are important fungal pathogens of maize and other cereals worldwide. In this study, we developed PCR-based protocols for the identification of these pathogens targeting the *gaoB* gene, which codes for galactose oxidase. The designed primers recognized isolates of *F. verticillioides* and *F. subglutinans* that were obtained from maize seeds from several producing regions of Brazil but did not recognize other *Fusarium* spp. or other fungal genera that were either obtained from fungal collections or isolated from maize seeds. A multiplex PCR protocol was established to simultaneously detect the genomic DNA from *F. verticillioides* and *F. subglutinans*. This protocol could detect the DNA from these fungi growing in artificially or naturally infected maize seeds. Another multiplex reaction with a pair of primers developed in this work combined with a pre-existing pair of primers has allowed identifying *F. subglutinans*, *F. konzum*, and *F. thapsinum*. In addition, the identification of *F. nygamai* was also possible using a combination of two PCR reactions described in this work, and another described in the literature.

## 1. Introduction

*Fusarium* species are important fungal pathogens of maize (*Zea mays* L.) and other cereals worldwide [1]. *Fusarium verticillioides* (Saccardo) Nirenberg (teleomorph: *Gibberella moniliformis*) is the fungus most commonly found associated with maize stem and ear-rot. This species belongs to mating population A of the species complex *Gibberella fujikuroi* (Sawada) Ito in Ito and K. Kimura, which has nine mating populations (A to I) with different toxicological profiles and preferential hosts [2]. *Fusarium subglutinans* (Wollenweber and Reinking) Nelson, Toussoun and Marasas (teleomorph: *Gibberella subglutinans*) belongs to mating population E of the *G. fujikuroi* species complex and is also a maize pathogen found mainly in cold regions of the world where maize is cultivated.

In addition to its importance in agriculture, *Fusarium* species can also cause several diseases in humans and animals because they produce harmful mycotoxins. The species *F. verticillioides* can produce significant amounts of fumonisins and other mycotoxins in maize grains. Fumonisins interfere with sphingolipid metabolism, and especially the isoform B1 (FB1) presents a great mycotoxicological concern because of its abundance in maize grains [3]. FB1 causes leukoencephalomalacia in horses, pulmonary edema in swine, poor performance in poultry, altered hepatic and immune function in cattle, and it has been associated with human esophageal cancer [3]. The species *F. subglutinans* produces low levels or no fumonisins but can produce other mycotoxins [1].

Seeds provide one of the most efficient methods of pathogen dissemination at great distances and allow pathogen introduction into new areas. In maize, both species *F. verticillioides* and *F. subglutinans* can be spread by seeds [1,4]. These pathogens reduce germination by seed decay, damping-off, and seedling blight. The extent to which maize seed contamination can be reduced is dependent upon the development of an efficient screening system. Such a screening system can also be of great utility in research programs aimed at expanding the knowledge of *Fusarium* disease epidemiology and in the selection of resistant maize genotypes.

The methods currently employed for the identification and differentiation of *F. verticillioides* and *F. subglutinans* from the other *Fusarium* species are based mainly on the morphology. Fertility tests with mating test lineages are necessary for specific identification. These methods are relatively simple and cheap in terms of the materials used, but they can be laborious and it may take weeks to obtain results. Furthermore, these methods are highly dependent on the analyst’s expertise. Molecular techniques, such as the polymerase chain reaction (PCR), are also used for species detection and identification of the *Fusarium* species genera [5]. PCR is a fast, sensitive, and very specific technique. Indeed, this technique has yielded great perspectives in seed pathology because only a small quantity of DNA is required to confirm the pathogen’s identity and its presence in host tissues [6]. There are several reports in the literature about the use of PCR for the detection of *F. verticillioides*, *F. subglutinans*, and *Fusarium proliferatum* [7–19]. However, almost all primer pairs developed so far for the identification of *F. verticillioides* and *F. subglutinans* had cross-reactivity with other species, and some of them could identify only toxigenic strains. Moreover, there is no method for the detection of some of the *G. fujikuroi* species that are pathogenic to sorghum (*Sorghum bicolor*, L. Moench), such as *Fusarium thapsinum* and *Fusarium nygamai*.

The galactose oxidase *gaoA* gene [20] has been used as a target for primers in PCR reactions for the specific detection of *Fusarium graminearum* (teleomorph: *Gibberella zeae*) [21,22]. Galactose oxidase is a copper enzyme that catalyzes the oxidation of primary alcohols to aldehydes with the concomitant reduction of O_2_ to H_2_O_2_ [23]. Three ortholog lineages of this gene have been identified in *Fusarium* and the lineage *gaoB* has been cloned in our laboratory from *F. verticillioides* and from *F. subglutinans* [24]. The *gaoB* lineage lacks introns and is 2040 bp in length [24].

Considering that *F. verticillioides* and *F. subglutinans* are important pathogens of maize and that they are transmitted by seeds, we have reasoned that it would be very important to develop a specific, reliable, and useful molecular diagnostic method for the detection of these pathogens in maize seeds. Thus, the objectives of the present work were: (i) to develop a PCR method for the molecular detection of *F. verticillioides* and *F. subglutinans* with primers targeting the recently cloned *gaoB* gene from those pathogens; and (ii) to use these primers in a multiplex PCR to simultaneously detect both species.

## 2. Results and Discussion

### 2.1. DNA Amplification and Primer Sensitivity Analysis

**Figure 1:**
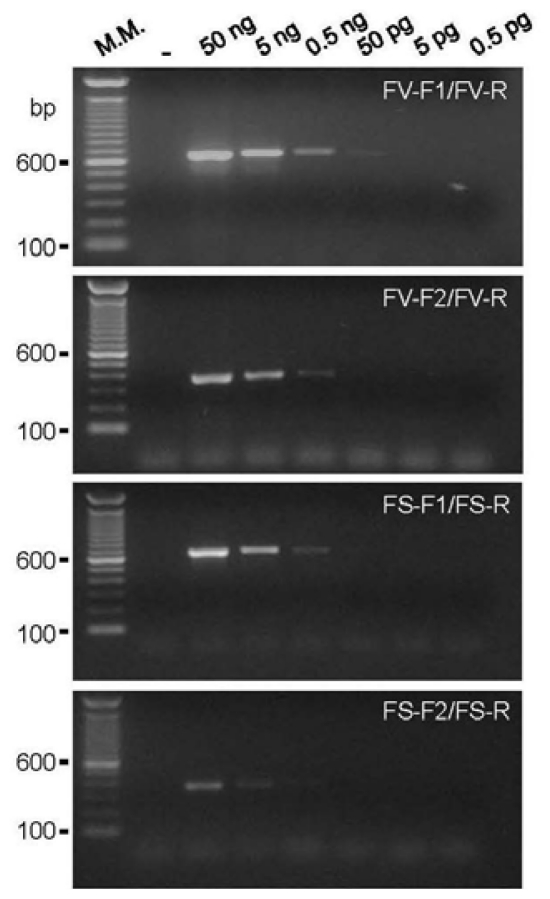
Sensitivity analysis. Electrophoresis on a 1.5% agarose gel stained with ethidium bromide showing the PCR reactions for the genomic DNA of *F. verticillioides* CML 767 with a 10-fold serial dilution for the FV primer pairs of and from *F. subglutinans* UnB 379 also with a 10-fold serial dilution for the FS primer pairs. M.M. indicates the 100-bp molecular marker (Invitrogen, USA). The negative sign (−) indicates the negative control reaction with no DNA added.

All of the new primer pairs amplified a unique band of the expected size with the genomic DNA from the control strains *F. verticillioides* (CML 767) or *F. subglutinans* (UnB 379). The lowest amount of genomic DNA of the control isolates that could generate a visible band in a conventional agarose gel stained with ethidium bromide was 50 pg (0.05 ng) for the primer pair FV-F1/FV-R and 500 pg (0.5 ng) for the other new primer pairs (Figure 1). This sensitivity is similar as that of other primers described in the literature [13,14,25].

### 2.2. Primer Specificity Analysis

**Figure 2:**
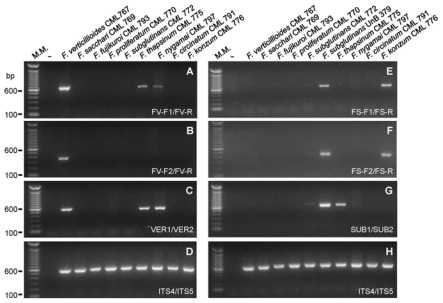
Specificity analysis of the primer pairs FV and FS described in this work and the primer pairs VER1/VER2 and SUB1/SUB2 described by Mulè *et al.* [14] with the *G. fujikuroi* species complex isolates genomic DNA. The PCR reactions were analyzed in 1.5% agarose gel stained with ethidium bromide. M.M. indicates the 100-bp molecular marker from Invitrogen (USA). (−) indicates the negative control reaction in which no DNA was added.

Regarding to the specificity analysis with the *G. fujikuroi* species, the primer pair FV-F1/FV-R targeting the *gaoB* gene from *F. verticillioides* amplified a DNA fragment of the expected size from the genomic DNA from *F. verticillioides*, but also from *F. thapsinum* and *F. nygamai*, two species phylogenetically related to *F. verticillioides* [2] (Figure 2A). The primer pair FV-F2/FV-R amplified a DNA fragment only from the *F. verticillioides* genomic DNA (Figure 2B). The two FS primer sets targeting the *gaoB* gene of *F. subglutinans* have amplified a DNA fragment of the expected size from the genomic DNA from the *F. subglutinans* strains used as control (UnB 379 and CML 772) and from *F. konzum* (Figure 2E,F), a species phylogenetic related to *F. subglutinans* [1]. The primer pair VER1/VER2 described by Mulè *et al.* [14] and targeting the calmodulin gene had specificity similar to the primer pair FV-F1/FV-R (Figure 2C). The primer set SUB1/SUB2 described by Mulè *et al.* [14] had a slightly different specificity of the FS primers, amplifying a DNA fragment from the genomic DNA from *F. subglutinans* and also from *F. thapsinum* (Figure 2G). The specificity of these primer pairs described by Mulè *et al.* [14] to the *G. fujikuroi* isolates had not been tested before.

**Figure 3:**
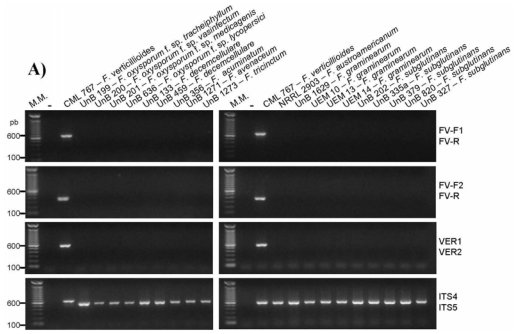

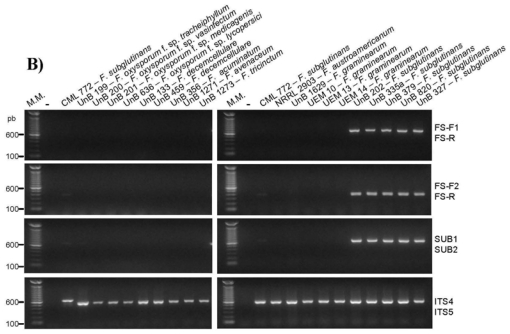
Specificity analysis with the isolates of *Fusarium* spp. genomic DNA. (**A**) FV (this work) and VER1/VER2 [14] primer pairs. (**B**) FS (this work) and SUB1/SUB2 [14] primer pairs. The PCR reactions were analyzed in 1.5% agarose gels stained with ethidium bromide. M.M. indicates the 100-bp molecular marker from Invitrogen (USA). (−) indicates the negative control reaction.

All designed primer pairs were unable to amplify any DNA fragment, specific or nonspecific, from the genomic DNA of several species of the *Fusarium* genera and from several other fungal genera that could be associated with maize seeds (Table 1, Figure 3). In addition, the FV-F2/FV-R and FS primer pairs were also unable to recognize any target in the genomic DNA of several fungi that were isolated from maize seeds in this work as *Penicillium purpurogenum*, *Penicillium digitatum*, *Penicillium* sp., *Stenocarpella* sp., *Curvularia* sp., *Cladosporium* sp., *Phlebiopsis* sp., *Pichia* sp., *Rhyzopus* sp., *Phoma* sp., *Thrichoderma* sp., *Epicocum* sp., *Cladosporium* sp., *Irpex* sp., *Microdochium nivale*, *Epicocum* sp., *Mucor* sp., *Fusarium graminearum*, *Aspergillus oryzae*, *Aspergillus candidus*, *Aspergillus flavus*, and *Aspergillus niger*.

Concerning the primer sensitivity testing against *Fusarium* isolates found in maize seeds, the two FV primer pairs were able to amplify a DNA fragment from all 47 *F. verticillioides* isolates obtained from maize seeds (Table 2). The FS primer pairs were also able to molecularly identify the obtained *F. subglutinans* isolates. The DNA of other found *Fusarium* species was not recognized by the new primer pairs (Table 2). The genomic DNA of the isolate RV27-2 resulted in positive PCR reactions for the primer pairs FV-F1/FV-R and VER1/VER2 [14], indicating that it could be *F. verticillioides*, *F. nygamai*, or *F. thapsinum*. As it resulted in a negative PCR reaction with the more specific FV-F2/FV-R primer pair, it could not be *F. verticillioides*. In addition, it also resulted in a negative PCR reaction with the SUB1/SUB2 [14] primer pair, demonstrating that it is neither *F. thapsinum* nor *F. subglutinans*. Thus we have concluded that this isolate is *F. nygamai*, what was confirmed in the Fusarium-ID analysis. The identification of the isolate RV 27-2 as *F. nygamai* points out that the combination of individual PCR reactions with the primers FV-F2/FV-R (this work), FV-F1/FV-R (this work), and SUB1/SUB2 [14] can be used for the identification of *F. nygamai*. The information that the FV and FS primer pairs do not recognize the *gaoB* gene of *F. andiyazi* (Table 2) enhances the data about their specificity.

### 2.3. Multiplex PCR Reactions

**Figure 4:**
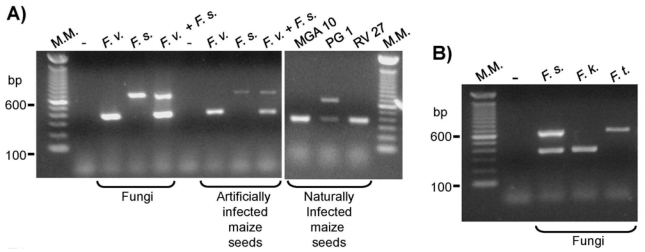
Multiplex PCR reactions. (**A**) A multiplex PCR reaction for the simultaneous detection of *F. verticillioides* and *F. subglutinans*. The PCR reactions were performed with FV-F2/FV-R and FS-F1/FS-R primer pairs and the genomic DNA of only *F. verticillioides* (50 ng), only *F. subglutinans* (50 ng), or of *F. verticillioides* (25 ng) together with *F. subglutinans* (25 ng). The genomic DNA (50 ng) extracted from mycelia of fungi growing on the top of and around cultured maize seeds that were artificially or naturally contaminated (MGA 10, PG 1, and RV 27 seed lots) was also used in the reaction. (**B**) The multiplex reactions with primer pairs FS-F2/FS-R and SUB1/SUB2 [14] to identify *F. subglutinans*, *F. thapsinum*, and *F. konzum*. M.M. indicates the 100-bp molecular marker from Invitrogen (USA). (−) indicates the negative control reaction in which no DNA was added. *F. v.* is *Fusarium verticillioides* CML 767. *F. s.* is *F. subglutinans* UnB 379. *F. k.* is *F. konzum* CML 776. *F. t.* is *F. thapsinum* CML 775.

The combination of the primer pairs FV-F2/FV-R and FS-F1/FS-R in only one PCR reaction could amplify a 370 bp DNA fragment from the *F. verticillioides* genomic DNA and a 649 bp DNA fragment from *F. subglutinans* genomic DNA (Figure 4A), what is consistent with the amplicon sizes obtained with the primer pairs used individually (Figure 1). In addition, this established reaction could also amplify DNA fragments of the expected size from genomic DNA extracted from fungi growing on and on the medium around the maize seeds that were artificially or naturally contaminated with *F. verticillioides* or *F. subglutinans*, or both species (Figure 4A). The naturally contaminated maize seed lots chosen for this analysis were: MGA 10 from which *F. verticillioides* was isolated, PG 1 from which *F. subglutinans* was isolated, and RV 27 from which neither of these fungi was initially isolated. The results obtained (Figure 4A) indicate the presence of *F. subglutinans* in the PG 1 sample and the presence of *F. verticillioides* in all three naturally contaminated seed samples tested, what is in accordance with the literature that reports the presence of *F. verticillioides* in 100% of the maize seeds in Brazil [26]. Möller *et al.* [13] have also set up a multiplex PCR to detect *F. verticillioides* and *F. subglutinans* simultaneously, but there are no data on the specificity of these primers with the genomic DNA of *F. circinatum*, *F. konzum*, and *F. andiyazi*. The present multiplex PCR reaction represents a significant advance for the simultaneous molecular detection of *F. verticillioides* and *F. subglutinans*.

When the primer pair FS-F2/FS-R was used in a multiplex reaction with the primer pair SUB1/SUB2 [14], two DNA fragments were amplified from the genomic DNA of *F. subglutinans*: one of 370 bp and one of 631 bp (Figure 4B). In addition, this set of primers could amplify a single DNA fragment of 370 bp when the genomic DNA from *F. konzum* was present in the reaction or a single DNA fragment of 631 bp when the genomic DNA from *F. thapsinum* was used in the reaction (Figure 4B). This reaction represents an improvement to the described reactions designed to detect *F. subglutinans*. The primers developed by Möller *et al.* [13] to identify *F. subglutinans* were not tested for all *G. fujikuroi* isolates, and the primers designed by Zheng and Ploetz [19] were specific for *F. subglutinans* and *F. nygamai*. In addition, there are few data in the literature about reactions to identify *F. thapsinum*, which is an important sorghum pathogen.

**Figure 5:**
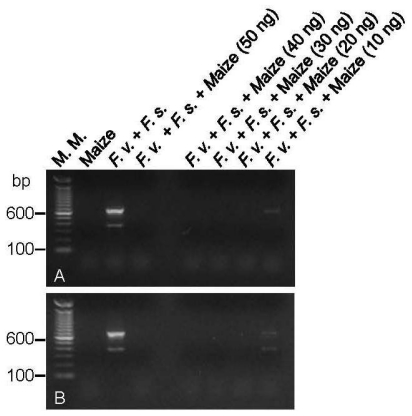
Multiplex PCR reactions for the simultaneous detection of *F. verticillioides* and *F. subglutinans* in the presence of maize DNA. The PCR reactions were performed with FV-F2/FV-R and FS-F1/FS-R primer pairs and the genomic DNA of maize (50 ng), of *F. verticillioides* together with *F. subglutinans* (0.5 ng of each in A and 5 ng of each in B) alone or with increasing amounts of maize DNA. M.M. indicates the 100-bp molecular marker from Invitrogen (USA). *F. v.* is *Fusarium verticillioides* CML 767. *F. s.* is *F. subglutinans* UnB 379.

The isolation of DNA directly from the seeds, without previous culture to obtain mycelia, was also tried but resulted in no DNA amplification in the multiplex PCR reaction to detect *F. verticillioides* and *F. subglutinans*. This could have happened because the amount of fungal cells in the contaminated seeds is too low or because of the presence of inhibitors in the extracted DNA. To test for the presence of inhibitors in the DNA extracted from maize, the established multiplex PCR reaction to simultaneously detect *F. verticillioides* and *F. subglutinans* was performed with the lowest amount of fungi DNA that could result in amplification spiked with increasing amounts of genomic DNA extracted from healthy maize seeds. The results shown in Figure 5 evidenced that maize DNA only did not inhibit the reaction when present in a low amount.

## 3. Experimental Section

### 3.1. Fungal Isolates

**Figure 6:**
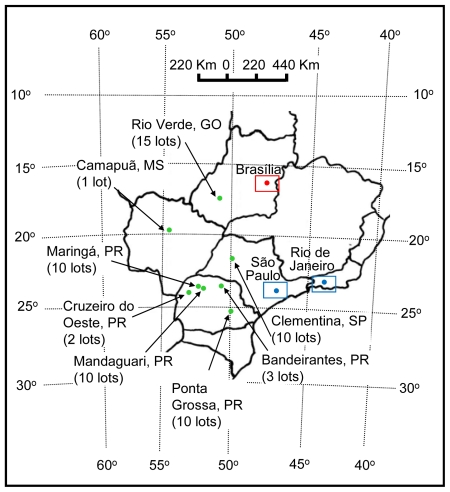
Map of Brazil indicating the cities and states where the maize seeds were obtained. The collected and analyzed number lots in each city are indicated. The largest cities in Brazil, São Paulo and Rio de Janeiro, and the capital, Brasília, are indicated.

The fungal isolates used in this work are listed in Table 1. To obtain a *Fusarium* collection from maize seeds, corn spikes showing signs of rot were collected from January to October of 2009, in fields in several producing regions of Brazil (Figure 6). The seeds of each spike were denominated as a lot and were kept in paper bags at 4 °C after insecticide treatment. A total of 57 maize seed lots were collected and analyzed. Six maize seeds of each lot, in duplicate, were disinfected by one min incubation in a solution containing 0.2% active chlorine, washed in sterile distilled water, and inoculated in a 10 mm diameter dish containing Malaquite Green Agar-MGA [27] supplemented with 350,000 UI/L of penicillin and 145 UI/L of streptomycin. The seeds plated in the medium were incubated for four to five days at 25 °C, with a photoperiod of 12 h. After germination, mycelia and conidia of peach or violet colored colonies were transferred to Petri dishes containing Carnation Leaf-piece Agar-CLA [1], which were incubated for a period of seven days at 25 °C, with a photoperiod of 12 h. A well-colonized carnation leaf fragment in the CLA culture was used for monosporic isolation as described in Nelson *et al.* [28]. The isolates morphologic characteristics were analyzed in micro culture performed in a one-cm^3^ block of Spezieller Nährstoffarmer Agar-SNA [1]. The isolates with cultural and morphological characteristics of *Fusarium* were cultivated for DNA extraction and molecular identification with the specific primers described by Mulè *et al.* [14]. These primers were chosen because they target a protein coding gene as the primers designed in this work. The genomic DNA of *Fusarium* isolates that were not identified as *F. verticillioides* or *F. subglutinans* were used in PCR reactions with specific primers for *F. proliferatum* [14]. These fungi were also analyzed using the methodology described in Geiser *et al.* [29] in which a portion of the elongation factor α was amplified through PCR, purified with the ExoSap-IT Kit (GE HealthCare, USA), and sequenced in the Center for the Human Genome Studies (CEGH) in the University of São Paulo (USP), Brazil. For the isolate identification, the obtained sequences were compared with sequences deposited in the data bank Fusarium-ID [29]. All obtained isolates are being maintained in the laboratory collection on PDA and SNA media with trimestral passages and in SNA medium under mineral oil.

To obtain fungi other than *F. verticillioides* or *F. subglutinans* from maize seeds, to be used in the primers specificity analysis, six maize seeds of each lot, in triplicate, were disinfected as described above and inoculated in a 10 mm diameter dish containing PDA, pH 4.5 with lactic acid [6], supplemented with penicillin and streptomycin, as described above. The seeds plated in the medium were incubated for five to seven days at 25 °C, with a photoperiod of 12 h. The fungi growing on and on the medium around the maize seeds with culture characteristics different from *G. fujikuroi* were submitted to monosporic isolation [28]. The classification of *Aspergillus* and *Penicillium* isolates was performed as described in Pitt and Hocking [30] with cultures in specific media. The molecular identification of the isolates of other genera was performed by the amplification of an rRNA gene fragment with the ITS4/5 primers [31], purification of the amplified DNA fragment with the PureLink™ PCR purification kit (Invitrogen, USA), sequencing in the CEGH (USP, Brazil) and comparison with sequences deposited in data banks. Specific identification of *F. graminearum* was performed in PCR reactions with the GOF/R specific primers [21]. All isolates obtained are being maintained in the laboratory collection on PDA with trimestral passages and on PDA medium under mineral oil.

### 3.2. Primer Design

**Figure 7:**
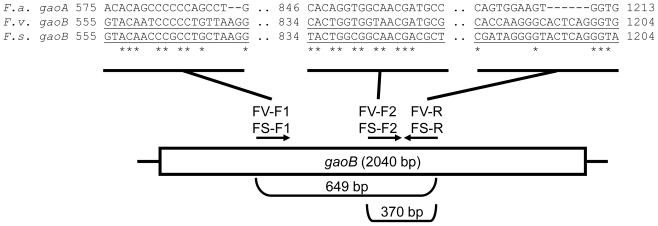
Primer design. The ClustalW alignment of portions of the galactose oxidase-coding *gaoA* gene from *F. austroamericanum* (*F. a.—*GenBank M86819) and the *gaoB* gene from *F. verticillioides* (*F. v.*—GenBank HM069186) and *F. subglutinans* (*F. s.*—GenBank HM069185) is shown above the positions of the designed primers regarding the *gaoB* gene from *F. verticillioides* and *F. subglutinans*. The primers sequences are underlined and the orientations are indicated by the arrows. The amplicon’s size is indicated on the bottom.

Initially, the *gaoB* genes from *F. verticillioides* and *F. subglutinans* [24] (GenBank AN HM069186 and HM069185, respectively) and the *gaoA* gene from *F. austroamericanum* [20] (GenBank AN M86819) were aligned with the ClustalW program. Low similarity regions were chosen for primer design. Four pairs of primers were designed; two directed to the *gaoB* gene from *F. verticillioides* (FV-F1/FV-R and FV-F2/FV-R) and two other for the *gaoB* gene from *F. subglutinans* (FS-F1/FS-R and FS-F2/FS-R) to amplify a DNA fragment of 649 bp and 370 bp, respectively (Figure 7).

The primer sensitivity was tested by a ten-fold serial dilution of the control isolates genomic DNA in the established PCR reactions. The primer specificity was tested in PCR reactions using genomic DNA from *G. fujikuroi* species isolates, other *Fusarium* spp., other fungal genera, and fungi isolated from maize seeds.

### 3.3. DNA Extraction

For the DNA extractions, an approximately one-cm^3^ fragment of a monosporic culture in inclined PDA was smashed and shaken in 5 mL of distilled water. Two mL of the obtained suspension were used as inoculum in Erlenmeyer flasks of 125 mL containing 25 mL of potato dextrose medium. These flasks were incubated for five days at 25 °C. The mycelia were collected by filtration in sterile gauze. The mycelia was macerated in a mortar with liquid nitrogen, and transferred to microcentrifuge tubes. The DNA was extracted from the macerated mycelia using the protocol described by Koenig *et al.* [32]. The DNA obtained was quantified by measuring the absorbance at 260 nm and/or by fluorometry using the Qubit Quantitation Fluorometer and the Quant-it™ dsDNA HS Assay Kit (Invitrogen, USA).

### 3.4. Polymerase Chain Reaction—PCR

The PCR with the primer pairs designed in this work was performed in a total volume of 25 μL of the following mixture: 1X enzyme buffer (20 mM Tris, pH 8.4, and 50 mM KCl), 1.5 mM MgCl_2_, 1.5 U of Platinum^®^ *Taq* DNA polymerase (Invitrogen, USA), 0.2 mM of each dNTP, 25 pmol of each primer (FW and RV), and 50 ng of the genomic DNA. The reaction consisted of 25 cycles of 1.5 min at 94 °C, 1.5 min at the recommended annealing temperature for each pair of primers (Table 3), and 2 min at 72 °C in a Techne Thermocycler (England). Before the cycles, the samples were heated for 5 min at 94 °C, and after the cycles, the samples were incubated for 10 min at 72 °C. Negative controls (no DNA template) were used in each experiment to test for the presence of DNA contamination of reagents and reaction mixtures. Ten microliters of the PCR reaction were analyzed in an agarose gel containing ethidium bromide (0.25 μg/mL). Molecular weight markers were the 100 bp markers from Invitrogen (USA). The PCR products were visualized and photographed under UV light. All genomic DNA that resulted in nonamplification with the other used primers was tested for PCR amplification quality with the primers ITS4/5 [31]. All the PCR with the other primer pairs used in this work was performed as above with the exception with the primer pair ef1/ef2 [29] that was performed in a total volume of 25 μL with 50 ng of genomic DNA template and following the authors’ methodology.

### 3.5. Multiplex PCR Reactions

A first multiplex PCR reaction to simultaneously detect *F. verticillioides* and *F. subglutinans* was established with the primer pairs FS-F1/FS-R and FV-F2/FV-R. Another multiplex PCR reaction to identify *F. subglutinans* was established with the primer pairs FS-F2/FS-R (this work) and SUB1/SUB2 [14]. Genomic DNA from *F. konzum* and *F. thapsinum* gave a positive signal in this last multiplex PCR reaction as well.

The reaction established to simultaneously detect *F. verticillioides* and *F. subglutinans* was also tested with genomic DNA extracted directly from artificially or naturally contaminated seeds or from fungi growing on artificially or naturally contaminated maize seeds using the protocol described by Koenig *et al.* [32]. To artificially contaminate maize seeds, healthy maize seeds were disinfected as described above and incubated for 5 min in 5 mL of a suspension of 10^4^ spores/mL of *F. verticillioides*, or *F. subglutinans*, and of *F. verticillioides* together with 10^4^ spores/mL of *F. subglutinans*. The artificially contaminated seeds were inoculated in Petri dishes containing MGA-penicillin-streptomycin medium [27], prepared as described above. These seeds were incubated for five days at 25 °C on the medium, with a period of 12 h of light. Naturally infected maize seeds (Ponta Grossa-1, Rio Verde 27, and Maringá-10) showing signs of rot were disinfected and incubated in the same way. The mycelia growing on the seeds and on the agar around the seeds were scraped and used for the DNA extraction that was conducted with the method described by Koenig *et al.* [32]. To test if maize DNA could inhibit the reaction, maize DNA was also extracted from health maize seeds using the protocol described by Koenig *et al.* [32] and this DNA was added in multiplex PCR reactions with DNA from the reference isolates of *F. verticillioides* and *F. subglutinans*.

The multiplex reactions were performed as shown above, except that they contained 25 pmol of each primer. The annealing temperatures are listed in Table 3. The amplification of specific fragments was analyzed by running 10 μL of the PCR reaction on a 1.5% agarose gel that was stained with ethidium bromide (0.25 μg/mL).

## 4. Conclusions

In conclusion, in this work, primers to identify *F. verticillioides* and *F. subglutinans* were designed and tested in PCR reactions. Additionally, they could be combined in a multiplex reaction to simultaneously detect these two species in naturally and artificially contaminated maize seeds. Another multiplex reaction with a pair of primers developed in this work combined with a pre-existing pair of primers [14] has allowed identifying *F. subglutinans*, *F. konzum*, and *F. thapsinum*. In addition, the identification of *F. nygamai* was also possible using a combination of two different reactions described in this work and one described previously [14]. The results obtained in this work and the used experimental approaches have a great potential value for the molecular based identification of difficult fungal phytopathogens.

## Figures and Tables

**Table 1:** Fungal isolates used in this study and PCR results with the indicated primers.

Isolates	References	Primers					

		FV-F1 FV-R	FV-F2 FV-R	FS-F1 FS-R	FS-F2 FS-R	VER1 VER2	SUB1 SUB2

*Fusarium verticillioides* (MP A) *	CML 767 a / KSU 999	+	+	−	−	+	−
*Fusarium sacchari* (MP B) *	CML 769 a / KSU 3853	−	−	−	−	−	−
*Fusarium fujikuroi* (MP C) *	CML 793 a / KSU 1994	−	−	−	−	−	−
*Fusarium proliferatum* (MP D) *	CML 770 a / KSU 4853	−	−	−	−	−	−
*Fusarium subglutinans* (MP E) *	CML 772 a / KSU 0990	−	−	+	+	−	+
*Fusarium thapsinum* (MP F) *	CML 775 a / KSU 4094	+	−	−	−	+	+
*Fusarium nygamai* (MP G) *	CML 797 a / KSU 5111	+	−	−	−	+	−
*Fusarium circinatum* (MP H *	CML 791 a / KSU 10850	−	−	−	−	−	−
*Fusarium konzum* (MP I) *	CML 776 a / KSU 10653	−	−	+	+	−	−
*Fusarium oxysporum* f. sp. *tracheiphyllum*	UnB 199 b	−	−	−	−	−	−
*Fusarium oxysporum* f. sp. *vasinfectum*	UnB 200 b	−	−	−	−	−	−
*Fusarium oxysporum* f. sp. *medicagenis*	UnB 201 b	−	−	−	−	−	−
*Fusarium oxysporum* f. sp. *lycopersici*	UnB 636 b	−	−	−	−	−	−
*Fusarium decemcellulare*	UnB 133 b	−	−	−	−	−	−
*Fusarium decemcellulare*	UnB 459 b	−	−	−	−	−	−
*Fusarium acuminatum*	UnB 326 b	−	−	−	−	−	−
*Fusarium avenaceum*	UnB 1271 b	−	−	−	−	−	−
*Fusarium tricinctum*	UnB 1273 b	−	−	−	−	−	−
*Fusarium austroamericanum*	NRRL 2903 c / ATCC 46032	−	−	−	−	−	−
*Fusarium graminearum*	UnB 1269 b	−	−	−	−	−	−
*Fusarium graminearum*	UEM 10 d	−	−	−	−	−	−
*Fusarium graminearum*	UEM 13 d	−	−	−	−	−	−
*Fusarium graminearum*	UEM 14 d	−	−	−	−	−	−
*Fusarium subglutinans*	UnB 202 b	−	−	+	+	−	+
*Fusarium subglutinans*	UnB 335a b	−	−	+	+	−	+
*Fusarium subglutinans*	UnB 379 b	−	−	+	+	−	+
*Fusarium subglutinans*	UnB 820 b	−	−	+	+	−	+
*Fusarium subglutinans*	UnB 327 b	−	−	+	+	−	+
*Fusarium verticillioides*	CMI 112801 c / NRRL 2284	+	+	−	−	+	−
*Curvularia* sp.	UnB 64 b	−	−	−	−	−	−
*Phoma* sp.	UnB 614 b	−	−	−	−	−	−
*Glomerella* sp.	UnB 1067 b	−	−	−	−	−	−
*Penicillium chrysogenum*	CMI 37767 c / ATCC 10002	−	−	−	−	−	−
*Penicillium expansum*	c	−	−	−	−	−	−
*Penicillium brevicompactum*	c	−	−	−	−	−	−
*Peniclillium griseofulvum*	c	−	−	−	−	−	−
*Colletotrichum truncatum*	UEPG 14 c	−	−	−	−	−	−
*Cochliobolus* sp.	UnB 580 b	−	−	−	−	−	−
*Ascochyta pisi*	UnB 617 b	−	−	−	−	−	−
*Pyrenophora* sp.	UEPG 67 c	−	−	−	−	−	−
*Cylindrocladium scoparium*	UEPG 16 c	−	−	−	−	−	−
*Phomopsis* sp.	UnB 602 b	−	−	−	−	−	−
*Macrophomina phaseolina*	c	−	−	−	−	−	−
*Sordaria* spp.	UnB 37 b	−	−	−	−	−	−
*Pestalotia* sp.	UnB 754 b	−	−	−	−	−	−
*Alternaria alternata*	UnB 555 b	−	−	−	−	−	−
*Aspergillus flavus*	c	−	−	−	−	−	−
*Aspergillus fumigatus*	30R c	−	−	−	−	−	−
*Rhyzopus arrhyzus*	CMI 83711 c / ATCC 2456	−	−	−	−	−	−

a Dr. L. Pfenning, Federal University of Lavras, Brazil;

b Dr. J. C. Dianese, Brasília University, Brazil;

c Dr. C. Kemmelmeier, State University of Maringá, Brazil;

d Molecularly classified in a previous work [21];

* MP = *G. Fukikuroi* complex Mating Population. nt = non tested.

**Table 2:** *Fusarium* isolates obtained from maize seeds and the PCR results with the indicated primers.

Isolate	Geographic origin (City, State)	*Fusarium* species	Primers						

			FV-F1 FV-R	FV-F2 FV-R	FS-F1 FS-R	FS-F2 FS-R	VER1 VER2 *	SUB1 SUB2 *	PRO1 PRO2 *

MGA 2-2	Maringá, PR	*F. verticillioides*	+	+	−	−	+	nt	nt
MGA 5-1	Maringá, PR	*F. verticillioides*	+	+	−	−	+	nt	nt
MGA 6-1	Maringá, PR	*F. verticillioides*	+	+	−	−	+	nt	nt
MGA 9-2	Maringá, PR	*F. verticillioides*	+	+	−	−	+	nt	nt
MGA 10-1	Maringá, PR	*F. verticillioides*	+	+	−	−	+	nt	nt
MGA 17-2	Maringá, PR	*F. verticillioides*	+	+	−	−	+	nt	nt
MGA 19-2	Maringá, PR	*F. verticillioides*	+	+	−	−	+	nt	nt
MGA 42-1	Maringá, PR	*F. verticillioides*	+	+	−	−	+	nt	nt
MGA 45-1	Maringá, PR	*F. verticillioides*	+	+	−	−	+	nt	nt
MGA 49-2	Maringá, PR	*F. verticillioides*	+	+	−	−	+	nt	nt
MGI 1-1	Mandaguari, PR	*F. verticillioides*	+	+	−	−	+	nt	nt
MGI 3-2	Mandaguari, PR	*F. verticillioides*	+	+	−	−	+	nt	nt
MGI 5-2	Mandaguari, PR	*F. verticillioides*	+	+	−	−	+	nt	nt
MGI 6-1	Mandaguari, PR	*F. verticillioides*	+	+	−	−	+	nt	nt
MGI 7-1	Mandaguari, PR	*F. verticillioides*	+	+	−	−	+	nt	nt
MGI 10-2	Mandaguari, PR	*F. verticillioides*	+	+	−	−	+	nt	nt
MGI 18-1	Mandaguari, PR	*F. verticillioides*	+	+	−	−	+	nt	nt
MGI 19-2	Mandaguari, PR	*F. verticillioides*	+	+	−	−	+	nt	nt
MGI 20-2	Mandaguari, PR	*F. verticillioides*	+	+	−	−	+	nt	nt
PG-2-1	Ponta Grossa, PR	*F. verticillioides*	+	+	−	−	+	nt	nt
PG-3-1	Ponta Grossa, PR	*F. verticillioides*	+	+	−	−	+	nt	nt
PG-4-1	Ponta Grossa, PR	*F. verticillioides*	+	+	−	−	+	nt	nt
PG-5-2	Ponta Grossa, PR	*F. verticillioides*	+	+	−	−	+	nt	nt
PG-6-1	Ponta Grossa, PR	*F. verticillioides*	+	+	−	−	+	nt	nt
CO 2-2	Cruzeiro do Oeste, PR	*F. verticillioides*	+	+	−	−	+	nt	nt
CO 3-2	Cruzeiro do Oeste, PR	*F. verticillioides*	+	+	−	−	+	nt	nt
BAN 2-2	Bandeirantes, PR	*F. verticillioides*	+	+	−	−	+	nt	nt
BAN 4-2	Bandeirantes, PR	*F. verticillioides*	+	+	−	−	+	nt	nt
BAN 5-2	Bandeirantes, PR	*F. verticillioides*	+	+	−	−	+	nt	nt
CPÃ 1-1	Camapuã, MS	*F. verticillioides*	+	+	−	−	+	nt	nt
RV 8-1	Rio Verde, GO	*F. verticillioides*	+	+	−	−	+	nt	nt
RV 12-2	Rio Verde, GO	*F. verticillioides*	+	+	−	−	+	nt	nt
RV 14-1	Rio Verde, GO	*F. verticillioides*	+	+	−	−	+	nt	nt
RV 17-1	Rio Verde, GO	*F. verticillioides*	+	+	−	−	+	nt	nt
RV 21-2	Rio Verde, GO	*F. verticillioides*	+	+	−	−	+	nt	nt
RV 25-1	Rio Verde, GO	*F. verticillioides*	+	+	−	−	+	nt	nt
RV 26-1	Rio Verde, GO	*F. verticillioides*	+	+	−	−	+	nt	nt
RV 28-1	Rio Verde, GO	*F. verticillioides*	+	+	−	−	+	nt	nt
RV 29-2	Rio Verde, GO	*F. verticillioides*	+	+	−	−	+	nt	nt
CMA 2-1	Clementina, SP	*F. verticillioides*	+	+	−	−	+	nt	nt
CMA 3-1	Clementina, SP	*F. verticillioides*	+	+	−	−	+	nt	nt
CMA 4-1	Clementina, SP	*F. verticillioides*	+	+	−	−	+	nt	nt
CMA 6-2	Clementina, SP	*F. verticillioides*	+	+	−	−	+	nt	nt
CMA 7-2	Clementina, SP	*F. verticillioides*	+	+	−	−	+	nt	nt
CMA 8-1	Clementina, SP	*F. verticillioides*	+	+	−	−	+	nt	nt
CMA 9-1	Clementina, SP	*F. verticillioides*	+	+	−	−	+	nt	nt
CMA 10-2	Clementina, SP	*F. verticillioides*	+	+	−	−	+	nt	nt
PG-1-2	Ponta Grossa, PR	*F. subglutinans*	−	−	+	+	−	+	−
RV 23-2	Rio Verde, GO	*F. subglutinans*	−	−	+	+	−	+	−
PG-1-1	Ponta Grossa, PR	*F. circinatum*	−	−	−	−	−	−	−
RV 27-1	Rio Verde, GO	*F. andiyazi*	−	−	−	−	−	−	−
RV 27-2	Rio Verde, GO	*F. nygamai*	+	−	−	−	+	−	−
RV 18-1	Rio Verde, GO	*F. incarnatum-equiseti*	−	−	−	−	−	−	−
CMA 1-2	Clementina, SP	*F. incarnatum-equiseti*	−	−	−	−	−	−	−
CMA 5-1	Clementina, SP	*F. incarnatum-equiseti*	−	−	−	−	−	−	−

nt = non tested.

* = Mulè *et al.* [14].

**Table 3:** Used primers.

Primer code	Sequence	Reference	Annealing temperature

VER1	5′-CTTCCTGCGATGTTTCTCC	Mulè *et al.* [14]	56 °C
VER2	5′-AATTGGCCATTGGTATTATATATCTA		

SUB1	5′-CTGTCGCTAACCTCTTTATCCA	Mulè *et al.* [14]	56 °C
SUB2	5′-CAGTATGGACGTTGGTATTATATCTAA		

PRO1	5′-CTTTCCGCCAAGTTTCTTC	Mulè *et al.* [14]	56 °C
PRO2	5′-TGTCAGTAACTCGACGTTGTTG		

ITS4	5′-TCCTCCGCTTATTGATATGC	White *et al.* [31]	50 °C
ITS5	5′-GAAGTAAAAGTCGTAACAAGG		

FV-F1	5′-GTACAATCCCCCTGTTAAGG	This work	62 °C
FV-R	5′-CACCCTGAGTGCCCTTGGTG		

FV-F2	5′-CACTGGTGGTAACGATGCG	This work	64 °C
FV-R	5′-CACCCTGAGTGCCCTTGGTG		

FS-F1	5′-GTACAACCCGCCTGCTAAGG	This work	62 °C
FS-R	5′-TACCCTGAGTACCCCTATCG		

FS-F2	5′-TACTGGCGGCAACGACGCT	This work	62 °C
FS-R	5′-TACCCTGAGTACCCCTATCG		

ef1	5′-ATGGGTAAGGA(A/G)GACAAGAC	Geiser *et al.* [29]	60 °C
ef2	5′-GGA(G/A)GTACCAGT(G/C)ATCATGTT		

GOFW	5′-ACCTCTGTTGTTCTTCCAGACGG	Biazio *et al.* [21]	55 °C
GORV	5′-CTGGTCAGTATTAACCGTGTGTG		

FV-F2	5′-CACTGGTGGTAACGATGCG	This work	64 °C
FV-R	5′-CACCCTGAGTGCCCTTGGTG		
FS-F1	5′-GTACAACCCGCCTGCTAAGG	This work	
FS-R	5′-TACCCTGAGTACCCCTATCG		

FS-F1	5′-TACTGGCGGCAACGACGCT	This work	56 °C
FS-R	5′-TACCCTGAGTACCCCTATCG		
SUB1	5′-CTGTCGCTAACCTCTTTATCCA	Mulè *et al.* [14]	
SUB2	5′-CAGTATGGACGTTGGTATTATATCTAA		
